# Anti-adhesive and pro-apoptotic effects of 2-hydroxyethyl methacrylate on human gingival fibroblasts co-cultured with *Streptococcus mitis* strains

**DOI:** 10.1111/j.1365-2591.2011.01935.x

**Published:** 2011-12

**Authors:** S Zara, M Di Giulio, S D’Ercole, L Cellini, A Cataldi

**Affiliations:** 1 Department of Drug Sciences, University “G. d’Annunzio” Chieti-PescaraChieti; 2 Department of Biomedical Sciences, University “G. d’Annunzio” Chieti-PescaraChieti, Italy; 3 Department of Medicine and Ageing Sciences, University “G. d’Annunzio” Chieti-PescaraChieti, Italy

**Keywords:** co-culture, HEMA, human gingival fibroblasts, *Streptococcus mitis*

## Abstract

**Aim:**

To evaluate and observe the cellular reactions that occur during the interaction/integration between 2-hydroxyethyl methacrylate/host tissue/microbial environment, in a co-culture of human gingival fibroblasts (HGF) and *Streptococcus mitis* strains.

**Methodology:**

*Streptococcus mitis* were cultured with strains in the presence of 3 mmol L^−1^ HEMA for 48 h and 72 h. Cytotoxicity was evaluated by the trypan blue dye exclusion test. Apoptosis was evaluated by TUNEL analysis. Adhesion was evaluated by immunofluorescence and western blot analyses. Quantitative analyses of the results were acquired by Qwin Plus 3.5 and QuantityOne I-D analysis software, respectively. The statistical significance of the results was evaluated using *t*-tests and linear regression tests.

**Results:**

The trypan blue dye test revealed 47.3% and 46.5% of dead fibroblasts after 48 and 72 h HEMA treatment, respectively, while bacterial viability was not influenced by the presence of HEMA and fibroblasts. The expression of pro-collagen I, involved in fibroblast adhesion, in untreated samples ranged from 12.49% to 6.91% of the positive area after 48 and 72 h, respectively, dropping to below 2% of the positive area in the other experimental conditions. Unlike the trypan blue test, co-cultured samples treated with HEMA showed 20% and 25% versus 17% and 21% (after 48 and 72 h, respectively) of apoptotic cells.

**Conclusions:**

The evidence for HEMA toxicity and anti-adhesive effects against eukaryotic cells was reduced in the presence of bacteria, suggesting that dental resins should be well polymerized to avoid the spread of toxic monomers within the mouth.

## Introduction

Composite materials consist of inorganic and organic components that during the application of light allow the unsaturated acrylic compounds to be converted into organic and inorganic matrices with a degree of conversion in the range of 55–65% (Miletic & Santini 2008). Because of the low degree of conversion, residual monomers of the composite materials can be eluted and swallowed (Uzunova *et al.* 2008) or diffused over time throughout the residual dentine (Costa *et al.* 1999), in microgram to milligram amounts (Volk *et al.* 2006). Furthermore, laboratory studies have revealed mutagenic, teratogenic and genotoxic effects of composite compounds (Schwengberg *et al.* 2005, Schweikl *et al.* 2006, Di Pietro *et al.* 2008), along with tissue inflammation (Bouillaguet *et al.* 1996), oxidative cell damage (Spagnuolo *et al.* 2006, Volk *et al.* 2006), apoptosis (Spagnuolo *et al.* 2004) and inhibition of DNA and protein synthesis (Hanks *et al.* 1991).

2-Hydroxyethyl methacrylate (HEMA) is a key component of resin-based compounds, frequently found in aqueous eluates from polymerized dental biomaterials (Guertsen 2000). It plays a pivotal role during dentine impregnation of the adhesive system, its high water affinity producing flow into the collagen network of the dentine organic matrix, thus favouring infiltration and preventing collagen collapse. Because HEMA has a low molecular weight and high hydrophilicity, it could diffuse throughout the residual dentine and affect the viability of the underlying odontoblasts, altering cell division and activity (Bouillaguet *et al.* 1996, 1998) and inducing hypersensitivity reactions in the pulp in susceptible individuals (Pashley 1996). Moreover, several studies have demonstrated that HEMA can affect the differentiation of fibroblasts into odontoblasts to inhibit collagen I, osteonectin and dentine sialoprotein production, to reduce mineral nodule formation (About *et al.* 2005) and finally to cause fragmentation of DNA strands (Paranjpe *et al.* 2005).

The aim of this study was to evaluate cellular and tissue reactions to HEMA in terms of cytotoxicity, adhesion and apoptotic events, in an *in vitro* co-culture model of human gingival fibroblasts (HGF) and *Streptococcus mitis* strains, to better understand interaction/integration processes occurring between biomaterials, host tissue and microbial environment. The null hypothesis was that there would be no cellular and tissue responses to HEMA in an *in vitro* co-culture model of HGF and *Streptococcus mitis* strains.

## Materials and methods

### Bacterial strains

The *Streptococcus* strains used for the experiments were a reference strain *S. mitis* ATCC 6249 and a clinical isolate strain from saliva *S. mitis* DS12. Strains were cultured in Trypticase soy broth (TSB; Oxoid, Milan, Italy) plus 1% (w/v) sucrose overnight at 37 °C under anaerobic atmosphere; then, the broth cultures were diluted 1:10 (v/v) in DMEM that was antibiotic and serum free and refreshed for 2 h at 37 °C in an orbital shaker at 160 rpm in aerobic conditions. Subsequently, the broth cultures were adjusted to 0.5 McFarland, approximately corresponding to 1.5 × 10^8^ CFU mL^−1^ for *S. mitis* ATCC 6249 and 1.2 × 10^8^ CFU mL^−1^ for *S. mitis* DS12, and used for experiments.

The enumeration of CFU mL^−1^ was performed by plating serial dilutions of the refreshed broth cultures on Trypticase soy agar containing 5% defibrinated sheep blood and on Mitis-Salivarius agar and incubated at 37 °C for 24 h in anaerobic atmosphere and for another 24 h in aerobic atmosphere (AnaeroGen; Oxoid) for a total of 48 h.

### Culture of human gingival fibroblasts

Human gingival fibroblasts were obtained from fragments of healthy marginal gingival tissue from the retromolar area taken during surgical extraction of impacted third molars. Signed informed consent was obtained from the donors according to a protocol approved by the University of Bologna. The tissue fragments were immediately placed in Dulbecco’s modified Eagle’s medium (DMEM) for at least 1 h, rinsed thrice in phosphate-buffered saline solution (PBS), minced into small tissue pieces and cultured in DMEM, containing 10% foetal bovine serum (FBS), 1% penicillin and streptomycin and 1% fungizone. Cells were maintained at 37 °C in a humidified atmosphere of 5% (v/v) CO_2_. Cultured HGF following 4–8 passages were used. Cells were seeded into 96-well and 6-well culture plates (tissue-culture-treated plates, Nunc, EuroClone SpA, Life-Sciences-Division, Milan, Italy) with DMEM containing 10% FBS, penicillin and streptomycin.

### Co-culture preparation

When cells reached confluence, the medium was replaced by a fresh one containing HEMA (previously dissolved in absolute ethanol because it is not completely hydrophilic) and bacteria. Because preliminary studies demonstrated that 5 mmol L^−1^ can be considered HEMA TC50 after 24 h of treatment (Issa *et al.* 2004, Falconi *et al.* 2007), a lower HEMA concentration (3 mmol L^−1^) was chosen for this model. Ethanol concentration in the medium was checked and maintained under 0.3% to exclude ethanol cytotoxicity. Moreover, controls in the presence of ethanol and in the absence of HEMA were performed to exclude ethanol cytotoxicity (data not shown).

For the evaluation of the effect of HEMA on this co-culture model, several experimental conditions were prepared:

*HGF*: Human gingival fibroblasts
*HGF-HEMA:* HGF in the presence of HEMA.
*HGF/Sm DS12:* HGF in the presence of clinical isolated *S. mitis.*
*HGF-HEMA Sm DS12:* HGF in the presence of HEMA and clinical isolated *S. mitis.*
*HGF/Sm ATCC:* HGF in the presence of ATCC *S. mitis.*
*HGF-HEMA/Sm ATCC:* HGF in the presence of HEMA and ATCC *S. mitis.*

All specimens were incubated for 48 and 72 h at 37 °C in 5% CO_2_. After incubation, cells were washed with PBS, trypsinized and processed for trypan blue dye exclusion test, which selectively identifies dead fibroblasts in blue, which were counted in a Burker chamber.

### Viability test and light microscopy observations

After incubation, the planktonic phase of all cultures was removed by aspiration and each well washed twice with phosphate-buffered saline solution (PBS). Attached bacteria were examined for viability with Live/Dead kit (Molecular Probes Inc., Invitrogen, San Giuliano Milanese, Italy) as indicated by the manufacturer and visualized under a fluorescent Leica 4000 DM microscope (Leica Cambridge Ltd, Cambridge, UK). For bacterial counting, ten fields of view, randomly chosen for each slide, were examined. The counts were repeated independently by three microbiologists using image analysis software Leica Qwin Plus 3.5 (Leica Cambridge Ltd). Microscopic observation was repeated by three independent experiments.

### Immunofluorescence analysis

For pro-collagen I immune labelling, cells were incubated in 5% rabbit serum in PBS for 20 min at room temperature followed by a 45-min incubation in the presence of goat polyclonal pro-collagen I antibody (Santa Cruz Biotechnology, Santa Cruz, CA, USA) diluted 1:100 in PBS, 5% Tween-20 and 2% bovine serum albumin (BSA) for 1 h at 37 °C. Slides were washed in PBS and reacted for 45 min with fluorescein isothiocyanate (FITC)-conjugated anti-goat immunoglobulin (IgG) antibody (Sigma, Milan, Italy) diluted 1:50 in PBS, 5% Tween-20 and 2% BSA for 45 min at 37 °C. After several washes in PBS, slides have been mounted in glycerol-DABCO (1-4-diazabicyclo[2-2-2]octane) containing 5 μg mL^−1^ DAPI (4-6,diamidino-2-phenyl-indol; Santa Cruz Biotechnology) to counterstain nuclei. The negative control, performed by omitting the primary antibody, was not subjected to FITC staining. The labelled slides have been examined under a Leica DM 4000 microscope (Leica Cambridge Ltd) equipped with Leica DFC 320 Videocamera (Leica Cambridge Ltd) to acquire and analyse computerized images.

### Western blot analysis

Total cell lysates (20 μg) were subjected to electrophoresis on a 10% sodium dodecyl sulphate (SDS)–polyacrylamide gel and transferred to nitrocellulose membranes. Nitrocellulose membranes, blocked in 5% non-fat milk, 10 mmol L^−1^ Tris–HCl pH 7.5, 100 mmol L^−1^ NaCl and 0.1% (v/v) Tween-20, were probed with goat pro-collagen I antibody (Santa Cruz Biotechnology) and then incubated in the presence of the specific enzyme-conjugated IgG horseradish peroxidase. Samples were normalized by incubating the membranes with a mouse β-tubulin monoclonal antibody. Immunoreactive bands were detected with Super Signal West Dura Extended Duration Substrate (Thermo Scientific Rockford, IL, USA). Densitometric values, expressed as integrated optical intensity (IOI), have been estimated in a CHEMIDOC XRS System with the QuantityOne I-D analysis software (Biorad Laboratories Inc., Hercules, CA, USA).

### TUNEL analysis

Terminal deoxynucleotidyl-transferase-mediated dUTP nick end-labelling (TUNEL) is a method of choice for a rapid identification and quantification of apoptotic cells. DNA strand breaks, yielded during apoptosis, can be identified by labelling free 3′-OH termini with modified nucleotides in an enzymatic reaction. All steps were undertaken with FragEL DNA fragmentation Detection kit according to the manufacturer’s instructions (Calbiochem Merck, Cambridge, MA, USA). After two rinses in PBS, slides were dehydrated, mounted by using a permanent media and examined under a Leica DM 4000 microscope (Leica Cambridge Ltd) equipped with Leica DFC 320 Videocamera (Leica Cambridge Ltd) to acquire and analyse computerized images.

### Computerized morphometry measurements and image analysis

After digitizing the images deriving from immunohistochemical stained sections, a Leica Qwin Plus 3.5 Software System (Leica Cambridge Ltd) was used to evaluate pro-collagen I expression and positive nuclei revealed by TUNEL analysis. Image analysis of protein expression was performed through the quantification of thresholded areas for immunocytochemical fluorescent colour per ten fields of light microscope observation.

Qwin Plus 3.5 assessments were logged to Microsoft Excel and processed for Standard Deviations and graphics. The statistical significance of the results was evaluated using the t-test and the linear regression test. Results are expressed as mean ± SD. Values of *P* < 0.05 were considered significant.

## Results

**Figure 1:**
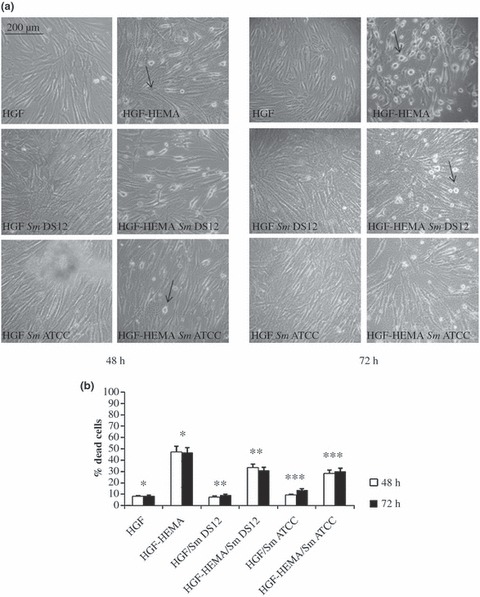
(a) Light phase-contrast microscopy images of co-cultured human gingival fibroblasts (HGF) cells in different experimental conditions: HGF, HGF-HEMA, HGF/*Sm* DS12, HGF-HEMA/*Sm* DS12, HGF/*Sm* ATCC and HGF-HEMA/*Sm* ATCC after 48 h (left panel) and 72 h (right panel) of treatment. Arrows indicate dead cells. Magnification 10×. (b) Graphic representation of cell death percentage assessed by trypan blue dye exclusion test, after 48 and 72 h of treatment. Data are the mean (±SD) of three different consistent experiments. *48 h HGF % dead cells versus 48 h HGF-HEMA % dead cells *P* < 0.01; *72 h HGF % dead cells versus 72 h HGF-HEMA % dead cells *P* < 0.01; **48 h HGF/Sm DS12% dead cells versus 48 h HGF-HEMA/Sm DS12% dead cells *P* < 0.05; **72 h HGF/Sm DS12% dead cells versus 72 h HGF-HEMA/Sm DS12% dead cells *P* < 0.05; ***48 h HGF/Sm ATCC % dead cells versus 48 h HGF-HEMA/Sm ATCC % dead cells *P* < 0.05; ***72 h HGF/Sm ATCC % dead cells versus 72 h HGF-HEMA/Sm ATCC % dead cells *P* < 0.05. Values of *P* < 0.05 have been considered significant.

**Figure 2:**
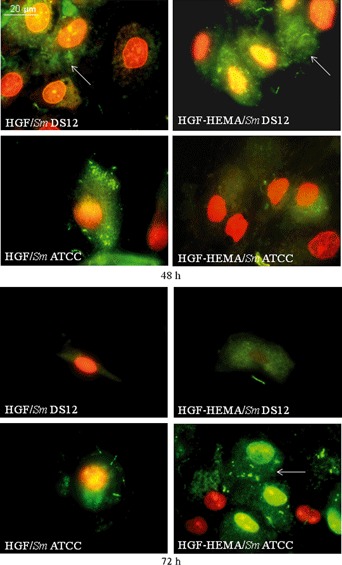
Bacterial viability, assessed by Live/Dead kit, of *Streptococcus mitis* strains co-cultured with human gingival fibroblasts (HGF) cells in different experimental conditions: HGF/*Sm* DS12, HGF-HEMA/*Sm* DS12, HGF/*Sm* ATCC and HGF-HEMA/*Sm* ATCC after 48 h (upper panel) and 72 h (lower panel) of treatment. Green fluorescence indicates viable bacteria (white arrow), and red fluorescence indicates dead bacteria (big red spots indicate HGF nuclei). Magnification 100×. Note that bacterial viability is high in all experimental conditions and does not seem to be influenced by HEMA and cells’ presence. Pictures are the most representative of three different consistent experiments.

Light phase-contrast pictures disclosed mostly viable and elongated fibroblasts either in the absence or in the presence of both *S. mitis* strains, whilst dead cells, appearing as round-shaped floating cells, were detected in HGF-HEMA, HGF-HEMA/*Sm* DS12 and HGF-HEMA/*Sm* ATCC 6249 samples after both 48 and 72 h (Fig. 1a). Trypan blue dye exclusion test quantified dead fibroblasts, confirming a physiological percentage of dead cells in HGF (8.1% and 8.4% after 48 and 72 h, respectively), HGF/*Sm* DS12 (7.5% and 9.2% after 48 and 72 h, respectively) and HGF/*Sm* ATCC 6249 (9.2% and 13.6% after 48 and 72 h, respectively) samples. As shown in Fig. 1b, an increase in the percentage of dead cells can be identified in the HGF/*Sm* ATCC samples, and percentages of dead cells reached 47.3% and 46.5% in HGF-HEMA after 48 and 72 h of treatment. Interestingly, after 48 h, HGF-HEMA/*Sm* DS12 and HGF-HEMA/*Sm* ATCC 6249 had 33.3% and 28.4% of dead cells, respectively, and after 72 h, 30.8% and 30% of dead cells, respectively. In parallel, bacterial viability evaluated by the Live/Dead kit revealed no significant differences between each condition detected (Fig. 2).

**Figure 3:**
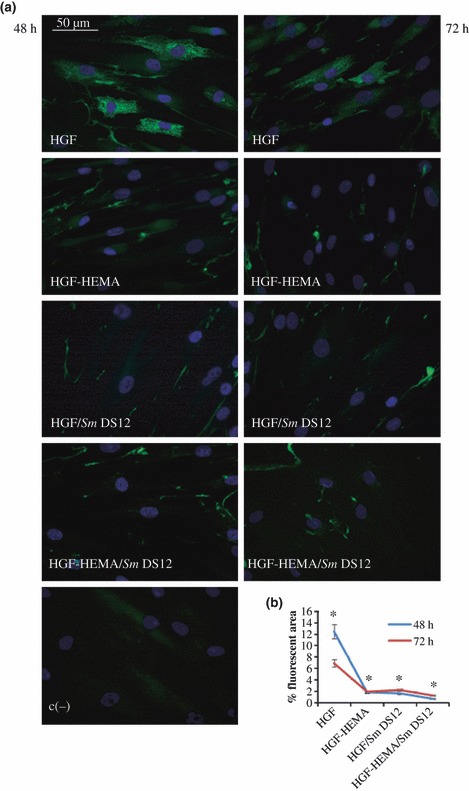
(a) Immunofluorescence analysis of pro-collagen I expression of co-cultured human gingival fibroblasts (HGF) cells in different experimental conditions: HGF, HGF-HEMA, HGF/*Sm* DS12, HGF-HEMA/*Sm* DS12 and negative control (c−) after 48 h (left panel) and 72 h (right panel) of treatment. Magnification 40×. Green fluorescence of fluorescein isothiocyanate (FITC) refers to pro-collagen I labelling; blue fluorescence of DAPI (4-6 diamino-2-phenyl-indol)-counterstains nuclei. (b) Graphic representation of densitometric analysis of pro-collagen-I-positive area (±SD) determined by direct visual counting of ten fields (mean values) for each of five slides per sample at 40× magnification. HGF shows a physiological pro-collagen I expression, labelling vanishes in all other experimental points. *48 h HGF % fluorescent area versus 48 h HGF-HEMA fluorescent area *P* < 0.01; *72 h HGF % fluorescent area versus 72 h HGF-HEMA fluorescent area *P* < 0.05; *48 h HGF % fluorescent area versus 48 h HGF/SmDS12 fluorescent area *P* < 0.05; *48 h HGF % fluorescent area versus 48 h HGF-HEMA/SmDS12 fluorescent area *P* < 0.05; *72 h HGF % fluorescent area versus 72 h HGF-HEMA/SmDS12 fluorescent area *P* < 0.05; Values of *P* < 0.05 have been considered significant.

**Figure 4:**
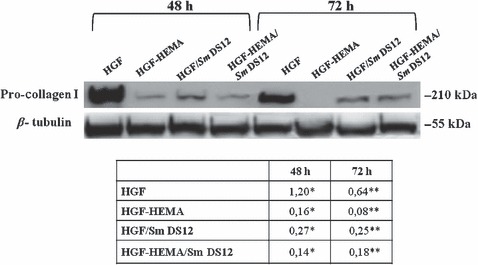
Western blot analysis of pro-collagen I expression. Each membrane was probed with anti-β-tubulin antibody to verify loading evenness. The most representative of three separate experiments is shown. *48 h human gingival fibroblasts (HGF) pro-collagen I versus 48 h HGF-HEMA pro-collagen I *P* < 0.05; **72 h HGF pro-collagen I versus 72 h HGF-HEMA pro-collagen I *P* < 0.05; *48 h HGF % pro-collagen I versus 48 h HGF/SmDS12 pro-collagen I *P* < 0.05; **72 h HGF pro-collagen I versus 72 h HGF/SmDS12 pro-collagen I *P* < 0.05; *48 h HGF pro-collagen I versus 48 h HGF-HEMA/SmDS12 pro-collagen I *P* < 0.05; **72 h HGF pro-collagen I versus 72 h HGF-HEMA/SmDS12 pro-collagen I *P* < 0.05; Values of *P* < 0.05 have been considered significant.

As the results derived from both the trypan blue dye exclusion test and the bacterial viability assay did not reveal any significant differences between the clinical *S. mitis* isolate and *S. mitis* ATCC, the *S. mitis* ATCC was excluded from the experimental protocol, thus concentrating attention on the effects exerted by the clinical bacterial strain about which little was known. The effects of HEMA and bacteria alone and in combination on the adhesion cell pro-collagen I protein expression, involved in structural adhesion events (Fox 2008), were evaluated by immunofluorescence microscopy analysis (Fig. 3a). HGF revealed a basal pro-collagen I level after both 48 and 72 h (12.49% and 6.91%, respectively), which decreased in HGF samples treated with HEMA co-cultured or not with bacteria, after 48 and 72 h as shown in Fig. 3b. To confirm imunocytochemical semi-quantitative data of pro-collagen I expression, a western blot analysis was performed (Fig. 4), disclosing a physiological pro-collagen I expression only in untreated samples (HGF), both after 48 and 72 h, and in parallel, a large decrease in molecule expression in the other specimens, according to the immunocytochemical results.

**Figure 5:**
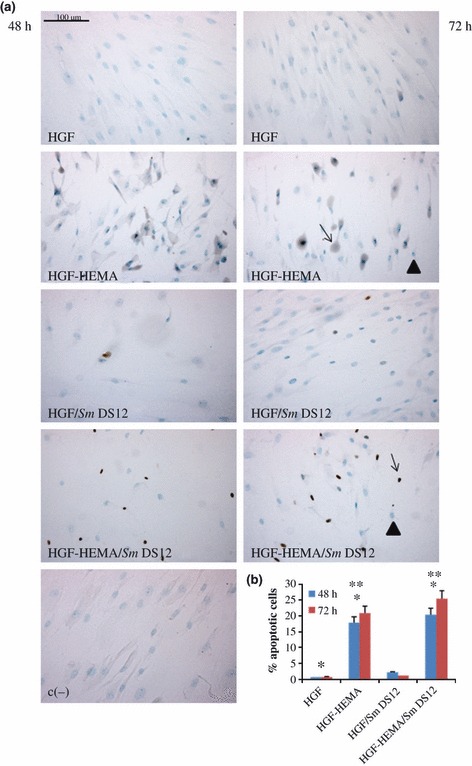
(a) TUNEL detection of co-cultured human gingival fibroblasts (HGF) apoptotic nuclei in different experimental conditions: HGF-HEMA, HGF/*Sm* DS12, HGF-HEMA/*Sm* DS12 and negative control (c−) after 48 h (left panel) and 72 h (right panel) of treatment. Blue staining and arrowheads indicate negative nuclei, and brown staining and arrows indicate positive nuclei. Magnification 20×. (b) Graphical representation of TUNEL analysis. Five slides have been examined per sample. Apoptotic cells have been counted out of a total cell number and expressed as percentage. Physiological levels of apoptotic cells can be detected in HGF and HGF/*Sm* DS12, and HGF-HEMA shows an increase in apoptotic nuclei percentage, more evident in HGF-HEMA/*Sm* DS12. Values represented in the graph are means (±SD) *n* = 3 for all groups. *48 h HGF % apoptotic nuclei versus 48 HGF-HEMA % apoptotic nuclei *P* < 0.01; *72 h HGF % apoptotic nuclei versus 72 h HGF-HEMA % apoptotic nuclei *P* < 0.01; *48 h HGF % apoptotic nuclei versus 48 HGF-HEMA/SmDS12% apoptotic nuclei *P* < 0.01; *72 h HGF % apoptotic nuclei versus 72 h HGF-HEMA/SmDS12% apoptotic nuclei *P* < 0.01; **48 h HGF-HEMA % apoptotic nuclei versus 48 h HGF-HEMA/SmDS12% apoptotic nuclei *P* < 0.05; **72 h HGF-HEMA % apoptotic nuclei versus 72 h HGF-HEMA/SmDS12% apoptotic nuclei *P* < 0.05. Values of *P* < 0.05 have been considered significant.

As a large decrease in adhesion could also induce a proliferative ability loss, along with apoptotic events, DNA fragmentation was evaluated by means of TUNEL analysis (Fig. 5a). The percentage of apoptotic nuclei shows physiological levels both in HGF and in HGF/*Sm* DS12 after 48 h (0.7% and 2.2%, respectively) and 72 h (0.8% and 1.2%, respectively) of treatment. HGF-HEMA sample discloses 17.9% and 21% of apoptotic cells after 48 and 72 h of treatment, respectively (Fig. 5b). Surprisingly and unlike that revealed by the trypan blue dye exclusion test, HGF-HEMA/*Sm* DS12 samples revealed 20.4% and 25.5% of apoptotic cells after 48 and 72 h, respectively, when compared with HGF-HEMA (17.9% and 21% after 48 and 72 h, respectively).

## Discussion

It has been demonstrated that unpolymerized monomers, such as HEMA, can be released by the polymerizable matrix (Durner *et al.* 2009, Teti *et al.* 2009) inducing several adverse effects, such as tissue inflammation (Bouillaguet *et al.* 1996), DNA fragmentation (Paranjpe *et al.* 2005), cell apoptosis (Spagnuolo *et al.* 2004) and protein synthesis inhibition (Hanks *et al.* 1991).

In the current study, the effects of a low concentration of HEMA at 48 and 72 h of exposure were analysed in primary HGF co-cultured with *S. mitis* clinical DS12 and ATCC 6249 bacterial strains to investigate prokaryotic and eukaryotic cell behaviour, in terms of viability, adhesion and apoptotic events. As toxicity of biomaterials influences the balance of cellular homeostasis, therefore affecting cellular responses, such as cell proliferation and morphological modifications, cell viability and death were evaluated through phase-contrast light microscope observation and the quantitative trypan blue dye exclusion test. The treatment with 3 mmol L^−1^ HEMA exerted a strong cytotoxicity for HGF, reaching almost 50% cell death after 48 h until 72 h, as already reported (Teti *et al.* 2009). However, previous studies have considered the oral cavity as a sterile environment without considering bacterial presence (Pawlowska *et al.* 2010, Sharrock & Grégoire 2010). To reproduce a condition close to the *in vivo* situation, the present set-up included a co-culture model of HGF and *S. mitis* bacterial strains. Phase-contrast light microscope observation and trypan blue dye test reveal that HGF, HGF/*Sm* DS12 and HGF/*Sm* ATCC had similar cell death trends underlining that *S. mitis* strains, at the tested concentrations, do not interfere with HGF viability. On the other hand, Sliepen *et al.* (2009) demonstrated a protective effect of a clinical *S. mitis* strain toward HGF when co-cultured for 24 h at 1 × 10^9^ CFU mL^−1^. Moreover, co-cultured samples, when treated with HEMA, disclosed an interesting effect of reduction in eukaryotic cell death with respect to HGF-HEMA samples. This result underlines the protective effect of *S. mitis* on HGF, against stress stimulus.

In parallel, prokaryotic cell viability was evaluated with the finding that *S. mitis* growth did not show significative differences at all experimental points, suggesting that its growth is not affected by HEMA and the presence of eukaryotic cells. As cell adhesion plays a critical role in cell morphology and function (Falconi *et al.* 2007, Tetè*et al.* 2009) and different stimuli can influence fibroblast adhesion (Liu *et al.* 2010), fibroblast expression of pro-collagen I, a molecule involved in adhesive processes, was analysed by immunohistochemical and western blot analyses. The data strongly support pro-collagen I expression reduction in HGF samples after 48- and 72-h HEMA treatment and show perinuclear localization of protein in control samples. Interestingly, bacterial presence (HGF/*Sm* DS12) caused a large decrease in pro-collagen I expression in co-cultured samples, comparable to the HGF-HEMA trend. This effect suggests that cell adhesion events could be affected not only by toxic stimuli such as HEMA spread but also by the presence of bacteria. The remarkable lowering of pro-collagen I expression, observed at all experimental points except the HGF sample, along with an high number of floating cells, supports the hypothesis that cells with reduced adherence can undergo an early death stimulus. TUNEL analysis, which can reveal early DNA breakdown within the nucleus, has confirmed this hypothesis showing low cell death percentages in HGF and HGF/*Sm* DS12 conditions and high cell death percentage in HGF-HEMA, according to the trypan blue dye exclusion test. TUNEL analysis has revealed a higher apoptotic cell percentage in HGF-HEMA/*Sm* DS12, when compared to HGF-HEMA, unlike the trypan blue dye test result, suggesting that HEMA has a toxic effect mainly inducing early cell necrosis rather than apoptosis. On the other hand, in the presence of bacteria, the high level of apoptosis observed could be considered a positive event that, by eliminating damaged cells, allows viable cells to perform a survival strategy against the toxic biomaterial. In any case, this hypothesis has to be confirmed by specific investigations that focus on the expression of molecules involved in apoptotic and survival events.

## Conclusion

The results demonstrated the toxic effect of HEMA and anti-adhesive effects against eukaryotic cells that was reduced by bacterial presence. This further supports the biological significance of bacteria within the oral cavity and suggests that dental restorative resins should be well polymerized to avoid toxic monomer spreading. However, more data should be gathered to confirm these findings to better understand the molecular mechanisms at the basis of such response.
